# Phoenixin as a New Target in the Development of Strategies for Endometriosis Diagnosis and Treatment

**DOI:** 10.3390/biomedicines9101427

**Published:** 2021-10-09

**Authors:** Karolina Iwona Kulinska, Mirosław Andrusiewicz, Anna Dera-Szymanowska, Maria Billert, Marek Skrzypski, Krzysztof Szymanowski, Ewa Nowak-Markwitz, Małgorzata Kotwicka, Maria Wołuń-Cholewa

**Affiliations:** 1 Chair and Department of Cell Biology, Poznan University of Medical Sciences, Rokietnicka 5D, 60-806 Poznan, Poland; andrus@ump.edu.pl (M.A.); mkotwic@ump.edu.pl (M.K.); 2 Clinic of Perinatology and Gynaecology, Poznan University of Medical Sciences, Polna 33, 60-535 Poznan, Poland; annaszerszen@wp.pl (A.D.-S.); kp.szymanowski@wp.pl (K.S.); 3 Department of Animal Physiology, Biochemistry and Biostructure, Poznan University of Life Sciences, Wolynska 35, 60-637 Poznan, Poland; maria.billert@gmail.com (M.B.); marek.skrzypski@up.poznan.pl (M.S.); 4 Chair and Department of Medical Education, Poznan University of Medical Sciences, Rokietnicka 7, 60-806 Poznan, Poland; 5 Gynecologic Oncology Department, Poznan University of Medical Sciences, Polna 33, 60-535 Poznan, Poland; ewanowakmarkwitz@gmail.com

**Keywords:** endometriosis, biomarkers, pain, G protein-coupled receptor 173 (GPR173), phoenixin (PNX), small integral membrane protein 20 (SMIM20)

## Abstract

Small integral membrane protein 20/phoenixin (SMIM20/PNX) and its receptor GPR173 (G Protein-Coupled Receptor 173) play a role in the regulation of the hypothalamic–pituitary–gonadal axis (HPG). The aim of the study was to determine PNX, FSH, LH, and 17β-estradiol association in women with endometriosis, and the expression of SMIM20/PNX signaling via GPR173. Serum PNX, FSH, LH, and 17β-estradiol concentrations were measured by enzyme and electrochemiluminescence immunoassay. SMIM20/PNX and GPR173 expression in the eutopic and ectopic endometrium was assessed by qPCR and immunohistochemistry. Reduced PNX level, increased LH/FSH ratio and elevated 17β-estradiol concentration were found in patients with endometriosis. No differences in *SMIM20* expression were observed between the studied endometria. *GPR173* expression was lower in ectopic than in eutopic endometria. SMIM20 expression was mainly restricted to stroma. GPR173 was detected in some eutopic and ectopic stromal cells and in eutopic glandular epithelial cells. Discriminant analysis indicates the diagnostic relevance of PNX and LH/FSH ratio in patients with endometriosis. In women with endometriosis, reduced PNX levels and GPR173 expression may be responsible for HPG axis dysregulation. These new insights may contribute to a better understanding of the pathophysiology of endometriosis and provide the basis for a new strategy for diagnosis and treatment of endometriosis.

## 1. Introduction

Endometriosis is one of the most common benign gynecological disorders. It is defined as the presence of stroma and glandular cells of the endometrium outside the uterine cavity [1]. It affects approximately 6–10% of women of reproductive age, increasing to 20–30% in women diagnosed with infertility [2]. Despite numerous studies, the etiology of endometriosis is still unknown. The most widely accepted theory of its formation is Sampson’s transplantation, and implantation theory, published a century ago. According to this theory, endometriosis results from retrograde menstrual reflux of endometrial fragments into the peritoneal cavity [3,4]. Noteworthy, most women have retrograde menstruation, and only a few develop endometriosis. Therefore, imbalance of the immune and endocrine systems, and the influence of environmental factors that ensure the implantation, growth, and persistence of the endometrium in ectopic sites are currently indicated [1,2,3,4,5].

The unclear etiology of endometriosis is the reason that a causal endometriosis treatment is essentially non-existent. Currently used pharmacotherapy masks the symptoms of the disease, often causing the occurrence of numerous side effects. This is sufficient to justify the search for new therapies, especially those that act causally.

Phoenixin (PNX) is a newly discovered neuropeptide synthesized by the paraventricular nuclei of the anterior hypothalamus. It positively regulates hypothalamic–pituitary–gonadal axis (HPG axis), reproductive system function, reduces cardiac reperfusion injury, modulates nutrition, improves memory, reduces pain and anxiety [6,7,8]. It occurs in two main isoforms: PNX-14 and PNX-20, formed from the transformation of the precursor protein small integral membrane protein 20 (SMIM20). As for biological activity, these isoforms do not differ [6]. Presumably, this neuropeptide, working via the G-protein-coupled receptor 173 (GPR173), activates the cAMP/PKA pathway, leading to cAMP-responsive element binding protein (CREB) phosphorylation (pCREB) [8].

In women, it has been shown that PNX can regulate the sexual cycle and ovarian follicle maturation and affect HPG axis by increasing hypothalamic (kisspeptin, GnRH) and pituitary (luteinizing hormone LH, follicle-stimulating hormone FSH) hormone production [7,9]. Decreased PNX-14 levels resulted in a delayed estrus phase in rats and a decreased GnRH receptor in the pituitary. Therefore, expression of PNX-14 is necessary for normal estrus cyclicity [7]. A positive correlation was observed between PNX-14 expression and LH, FSH, and testosterone and a negative correlation with serum estradiol and insulin levels in women with polycystic ovary syndrome (PCOS) [10]. In a rat model of PCOS, there was an increase in SMIM20 expression and PNX-14 synthesis in serum [11]. Moreover, GnRH analogs are often used to treat endometriosis and have been found to increase SMIM20 expression and decrease GPR173 receptor expression in the rat hypothalamus, pituitary, and ovaries [12]. It is worth noting that both SMIM20 and GPR173 are expressed in the human endometrium [13,14]. Currently, nothing is known about the role of PNX-14 in endometriosis.

The present study aimed to investigate whether patients with ovarian-localized endometriosis relative to patients without endometriosis have altered serum PNX and FSH, LH and 17β-estradiol levels. Expression of the SMIM20 precursor protein and the GPR173 receptor was also evaluated. We assume that the action of PNX outside the HPG axis is GnRH-dependent. In view of the reduced expression of the GnRH receptor, and GnRH itself, in the eutopic endometrium of women with endometriosis, altered expression of SMIM20 and the GPR173 receptor in the eutopic and ectopic endometrium of these women is expected.

## 2. Materials and Methods

### 2.1. Biological Materials

The study used archival material collected from 2008–2012: frozen sera, isolated RNA, and formalin-fixed paraffin-embedded endometrium. The age of patients included in the study ranged from 28 to 42 years and all had normal, regular menstrual cycles. The control group consisted of patients undergoing laparoscopy for non-endometrial pathologies (leiomyomata, cervical intraepithelial neoplasia, and benign ovarian cyst). The studied group consisted of women diagnosed with ovarian endometriosis (III/IV degree). Patients with endometriosis had not been treated pharmacologically for at least 6 months. Exclusion criteria for the studied group were gynecological malignant neoplasms and hormonal therapy. All women were operated on in the first phase of the menstrual cycle, between days 7th and 12th.

These preliminary classifications were then verified by histological analysis. Women with histological evidence of necrosis and active inflammation were excluded from the study.

Frozen sera were obtained from peripheral blood before surgery from the antecubital vein of 23 healthy volunteers and 32 patients diagnosed with endometriosis.

Archival RNA was isolated from eutopic and ectopic endometria obtained from 108 patients: 46 controls and 62 with ovarian endometriosis.

Formalin-fixed, paraffin-embedded tissues consisted of 15 eutopic and ectopic endometria.

### 2.2. Electrochemiluminescence Immunoassay (ECLIA)

FSH, LH and 17β-estradiol concentrations were measured by electrochemiluminescence immunoassay (Roche Diagnostics, Indianapolis, IN, USA) as described previously [15].

### 2.3. Enzyme-Linked Immunoassay (EIA)

PNX concentration was measured by fluorescent EIA kit (assay range 5.6–160 pg/mL) according to the manufacturer’s protocol (Phoenix Pharmaceuticals Inc., Burlingame, CA, USA). This kit has a 100% reactivity with PNX-14 and PNX-20.

All samples were assayed without any dilution. The final fluorescence intensity values were measured using a microplate reader (Synergy 2 Multi-Mode Microplate Reader, BioTek, Winooski, VT, USA) with the excitation and emission wavelengths of 325 and 420 nm, respectively.

### 2.4. SMIM20 and GPR173 Expression

#### 2.4.1. RNA Quality and Quantity Assessment

Archived RNA was used for qPCR analysis. The concentration of mRNA was measured spectrophotometrically (NanoPhotometer^®^ NP-80; IMPLEN, München, Germany). Quality and integrity of mRNA were assessed by gel electrophoresis in 1% agarose in FA buffer (200 mM 3-[N-morpholino]propanesulfonic acid (MOPS), 50 mM sodium acetate, 10 mM EDTA, and 2 mL of 37% formaldehyde per 100 mL of buffer; LabEmpire, Rzeszow, Poland). Samples with visible 18S and 28S bands were used for further qPCR analysis [16,17].

#### 2.4.2. Reverse Transcription

500 ng RNA (50 ng/µL) was reverse transcribed into cDNA according to the manufacturer’s protocol (Roche, Basel, Switzerland). The reaction mixture contained: 5 pm/µL of universal oligo(d)T_10_ primer, 1 pm/µL of random hexamer primer (Genomed, Warsaw, Poland), 0.5 U/µL Transcriptor Reverse Transcriptase, 0.25 U/µL RNase Inhibitor, 1X Reverse Transcriptase Buffer, 1 mM dNTPs (Roche, Basel, Switzerland), 0.1 U/µL E. coli poly(A)polymerase, and 0.1 mM dATP (Carolina Biosystems, Prague, Czech Republic). The RNA template, primers, and water mixture were incubated for 10 minutes at 65 °C and subsequently chilled on ice. After adding the remaining reaction compounds, the reaction was processed as described previously [16] and obtained cDNA was used immediately or stored at −20 °C.

#### 2.4.3. Real-Time PCR

mRNA expression of *SMIM20* and *GPR173* were assessed by quantitative PCR in a LightCycler 2.0 carousel glass capillary-based system (Roche, Manheim, Germany). Eva Green was used as a detection dye. The qPCR reaction mixture of 10 µL contained: 1X HotFire Pol Eva Green qPCR Mix Plus, 5 pm/µL of each genes’ specific forward and reverse primers (Table 1), and 2 µL cDNA. The following thermal profile was applied: pre-incubation step (12 minutes, 95 °C), followed by 45 cycles of amplification with the end-point acquisition (15 s at 95 °C, 30 s at 60 °C and 20 s at 72 °C for *SMIM20*, *B2M*, *GAPDH*, *HPRT1* or 15 s at 95 °C, 20 s at 55 °C and 6 s at 72 °C for *GPR173*). Amplification step was followed by a melting curve analysis at 95 °C for 0 sec and 65 °C for 15 s and 97 °C at the step acquisition mode (continuous fluorescence measurement; ramping rate, 0.1). Three reference genes: *beta-2-microglobulin (B2M)*, *glyceraldehyde-3-phosphate dehydrogenase (GAPDH)*, and *hypoxanthine phosphoribosyltransferase 1 (HPRT1)* were analyzed to choose the most stable. As a negative control, the reverse transcription reaction of mixed mRNAs without reverse transcriptase was used.

qPCR reactions were made in duplicates. Standard curves of each gene were performed to calculate the efficiency of the PCR reaction using serial dilutions of cDNA.

mRNA expression levels were normalized to the most stable reference gene and calculated using the relative quantification method. The concentration ratio (Cr) was used in the further analyses.

### 2.5. Immunohistochemistry

#### 2.5.1. SMIM20 Immunostaining

Slides were deparaffinized and rehydrated as mentioned above. After antigen retrieval and blocking in 2.5% goat serum, sections were incubated overnight at 4 °C with 1:500 polyclonal anti-SMIM20 antibody (ThermoFisher Scientific, Carlsbad, CA, USA). Next, slides were washed in TBS-T and stained with a secondary anti-rabbit DyLight 594-conjugated antibody. DAPI was used to detect nuclei. Imaging was performed using a Zeiss LSM 780 confocal microscopy system (Carl Zeiss Meditec AG, Jena, Germany). In all immunohistochemical negative control reactions, the primary antibody incubation step was omitted.

#### 2.5.2. PNX-14 and GPR173 Protein Co-Localization

Paraffin-embedded archival tissue samples were cut into 4 µm slides. After deparaffinization in xylene (65 °C, 30 minutes) and rehydration in decreasing alcohol concentrations (100%, 96%, 90%, 80%, 70%, 50%) and water, the sections were boiled in a microwave in sodium citrate buffer (pH 6.0, 3 × 5 minutes at 600 W; Agilent, Santa Clara, CA, USA) for antigen retrieval and rinsed in TBS-T buffer (100 mM Tris, 65 mM NaCl, 0.05% Tween-20, pH 7.5; Avantor Performance Materials Poland, Gliwice, Poland).

In the IHC reactions, first, slides were incubated in TBS-T buffer with 2.5% horse serum at room temperature for one hour to block the non-specific binding of the antibody. Next, sections were incubated in a humid chamber overnight at 4 °C with rabbit polyclonal anti-GPR173 antibodies (1:500; ThermoFisher Scientific, Waltham, MA, USA). Slides were then washed twice in TBS-T buffer (5 minutes) and incubated in darkness at room temperature for 1 h with a secondary horse anti-rabbit DyLight 488-conjugated antibody (Vector Laboratories, Inc., Burlingame, CA, USA).

After rinsing the unbounded antibodies three times in TBS-T buffer for 5 min and blocking in TBS-T buffer supplemented with 2.5% goat serum (Vector Laboratories, Inc., Burlingame, CA, USA), subsequently, second immunohistochemistry staining was performed. The slides were incubated overnight at 4 °C with polyclonal anti-PNX-14 antibody (1:500; Phoenix Pharmaceuticals, Inc., Burlingame, CA, USA). After washing in TBS-T buffer (2 × 5 minutes), slides were incubated with a secondary goat anti-rabbit DyLight 594-conjugated antibody (in the dark, room temperature, 1 h Vector Laboratories, Inc., Burlingame, CA, USA). Next, slides were washed 3X in TBS-T buffer and stained with 1 µg/mL DAPI at room temperature for 5 minutes (ThermoFisher Scientific, Carlsbad, CA, USA) to visualize the nuclei. Imaging was performed using a Zeiss LSM 780 confocal microscopy system (Carl Zeiss Meditec AG, Jena, Germany).

### 2.6. Statistical Analyses

Statistical analyses were performed using Statistica^®^ Version 13.5.0 software for Windows (TIBCO Software Inc., Palo Alto, CA, USA). The results were compared in groups: controls vs. cases. All continuous variables were checked for outliers and were winsorized if any were present using the equation (mean ± 2* standard deviations) [18]. The Shapiro–Wilk test was used for the normality of continuous variable distribution assessment. The median and interquartile range were used to describe experimental results. The differences in expression levels and serum concentration between the controls and cases were evaluated using the Mann–Whitney U test, and the results were described as median [Q1 (lower quartile)-Q3 (upper quartile)]. Associations between hormone levels were correlated using a nonparametric Spearman’s rank test. Moreover, using a system of linear discriminant equations, discriminant analysis of the serum hormone concentrations profile was performed. The patient was classified into the group for which the value of the equation was the highest. Data were considered statistically significant at *p* < 0.05. Reference gene expression stability was analyzed using the Microsoft Excel plugin NormFinder v0.953 (available at: https://moma.dk/normfinder-software, last accessed: 8 October 2021).

## 3. Results

### 3.1. Analysis of PNX, FSH, LH, 17β-Estradiol Serum Levels

The statistical analysis of PNX, FSH, LH and 17β-estradiol concentrations were presented in Table 2. The serum PNX concentration was significantly lower in the studied patients (cases) (Me = 76.7 pg/mL, [Q1-Q3] = [51.1–101.6]) than in controls (Me = 324.8 pg/mL, [Q1-Q3] = [214.9–399.3]). No significant differences in FSH and LH serum levels were observed between studied samples. Moreover, the ratio of LH to FSH concentrations was calculated and it was observed statistically significant higher ratio in women with endometriosis (Me = 1.3, [Q1-Q3] = [0.8–2.1]) compared to healthy women (Me = 0.5, [Q1-Q3] = [0.3–0.7]). A similar difference was found in the 17β-estradiol concentration analysis. The level was significantly higher in women with endometriosis (Me = 51.9 pg/mL, [Q1-Q3] = [35.1–153.2]) than in controls (Me = 21.3 pg/mL, [Q1-Q3] = [15.3–54.6]).

A strong positive correlation was observed between PNX and FSH levels in the control group (r = 0.52; *p* = 0.03) and a negative correlation between PNX-14 and 17β-estradiol concentrations (r = −0.56; *p* = 0.02)

The final discriminant analysis model used for the tested serum hormone levels includes the PNX concentration and the LH/FSH ratio. Table 3 shows the Wilks’ Lambda values, F test, and significance levels.

Based on the presented results, the proposed model including PNX level and LH to FSH ratio distinguishes sera of women with endometriosis from healthy women at a confidence level of up to 99.9%. The classification accuracy is 96% in the case of women with endometriosis and 67% in the case of healthy patients.

### 3.2. Real-Time PCR: SMIM20 and GPR173 Expression

**Figure 1:**
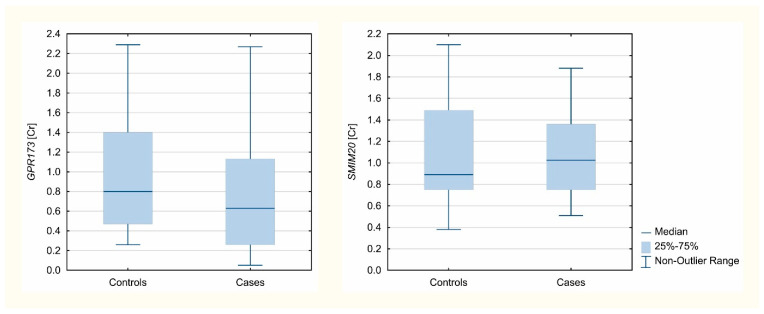
Expression level concentration ratios of analyzed genes normalized to *B2M*.

Three different reference genes were analyzed: *B2M*, *GAPDH*, and *HPRT1*. Accordingly, using NormFinder software, the most stable was *B2M* apart from tissue origin, and thus this gene was used for normalization and concentration ratio calculations. *GPR173* and *SMIM20* mRNA expression was confirmed in 104 and 108 analyzed samples, respectively. *GPR173* normalized mRNA expression was significantly lower in the studied patients (Me = 0.63; [Q1-Q3] = [0.26–1.13]) than in controls (Me = 0.8, [Q1-Q3] = [0.47–1.4]); (*p* = 0.0215). There was no difference observed between the groups in the case of the *SMIM20* normalized expression (*p* = 0.7702). The Mann–Whitney U test results are presented in Table 4. Analyzed gene expression Cr values, normalized to B2M, are presented in Figure 1.

Analyzed gene expression Cr values, normalized to *B2M*, are presented in Figure 1.

### 3.3. Immunohistochemistry SMIM20, PNX-14, and GPR173

**Figure 2:**
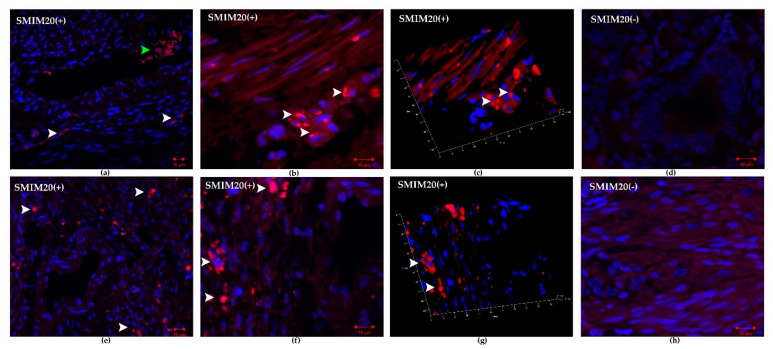
Immunohistochemical localization of SMIM20 in eutopic (**a**–**d**) and ectopic (**e**–**h**) endometrium. SMIM20 was mostly detected in the cytoplasm of several stromal cells. (**a**) control endometrium—SMIM20 was mostly detected in the cytoplasm of several stromal cells (white arrowheads), green arrowhead indicates erythrocytes; magnification 10×, (**b**) magnified image of immunopositive eutopic fibroblasts against SMIM20 (white arrowheads), magnification 40×, (**c**) 3D rendered image showing the presence of SMIM20 in eutopic stromal cells (white arrowheads); magnification 40×, (**d**) negative control of eutopic endometrium, magnification 40× (**e**) endometriosis—SMIM20 was mostly detected in the cytoplasm of several stromal cells (white arrowheads); magnification 10×, (**f**) magnified image of immunopositive ectopic stromal cells against SMIM20 (white arrowheads); magnification 40×; (**g**) 3D rendered image showing the presence of SMIM20 in ectopic stromal cells (white arrowheads); magnification 40× (**d**) negative control of ectopic endometrium, magnification 40×.

Figure 2 shows SMIM20 (red color) immunolocalization in eutopic (a–d) and ectopic (e–h) endometrium. SMIM20 is a precursor protein of PNX-14, and its expression was restricted mainly to the stromal cells (Figure 2).

**Figure 3:**
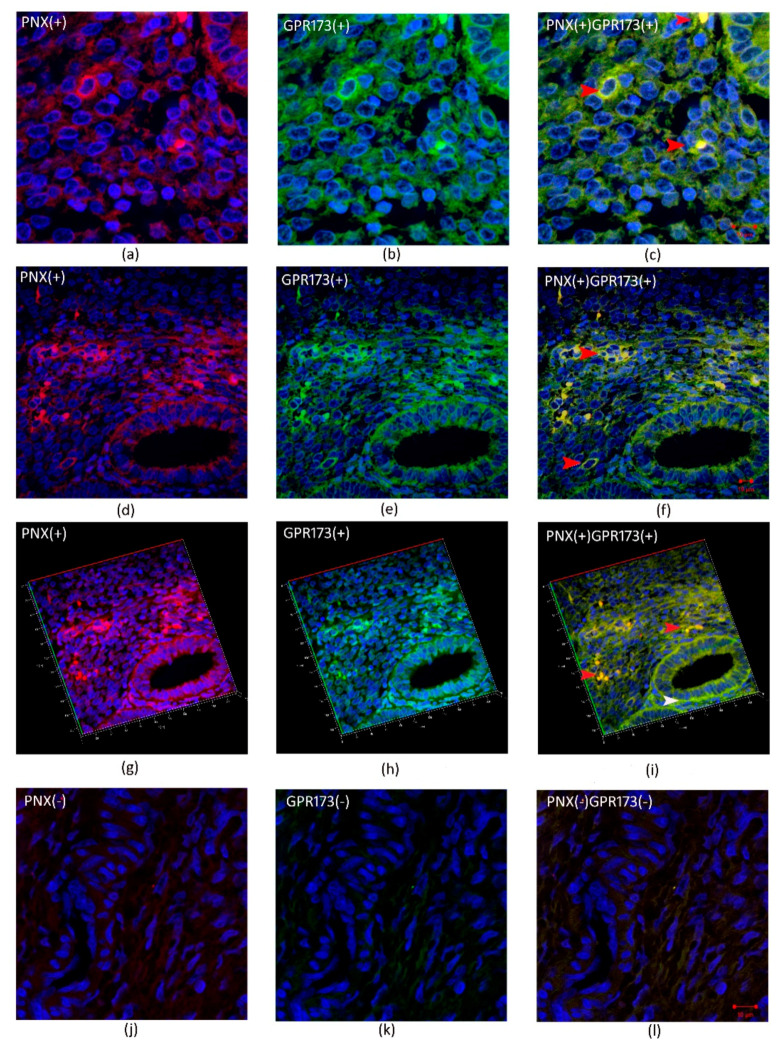
Immunohistochemical localization of PNX-14 and GPR173 in eutopic endometrium. (**a**,**d**,**g**)- show immunolocalization of PNX-14 in the cytoplasm of stromal cells of eutopic endometrium; (**j**)-negative control; (**b**,**e**,**h**)—show immunolocalization of GPR173 in the membrane of stromal cells; (**k**)-negative control and (**c**,**f**,**i**) and (**l**) are merged images showing co-localization of PNX-14 and GPR173. Red arrowheads indicate immunopositive stromal cells against PNX/GPR173; white arrowhead indicates GPR173-positive immunostaining glandular epithelial cells.

**Figure 4:**
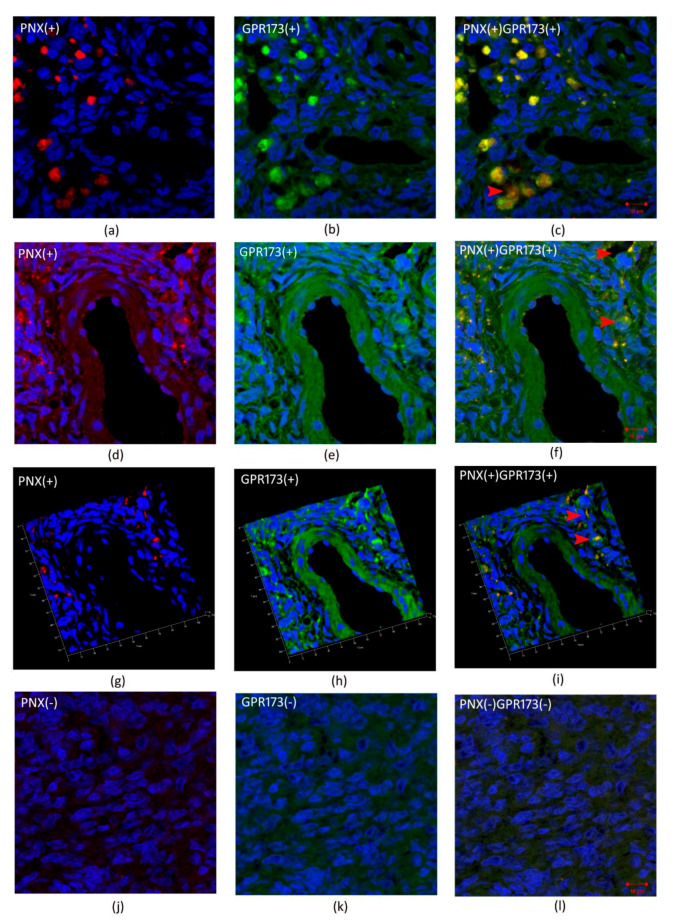
Immunohistochemical localization of PNX and GPR173 in ectopic endometrium. (**a**,**d**,**g**)- show immunolocalization of PNX-14 in the cytoplasm of stromal cells of ectopic endometrium; (**j**)-negative control; (**b**,**e**,**h**)—show immunolocalization of GPR173 in the membrane of stromal cells; (**k**)-negative control and (**c**,**f**,**i**) and (**l**) are merged images showing co-localization of PNX-14 and GPR173. Red arrowheads indicate immunopositive stromal cells against PNX/GPR173.

Figure 3 and Figure 4 show cleavage product of SMIM20—PNX-14 (red color) and GPR173 (green color) immunolocalization in eutopic (Figure 3) and ectopic (Figure 4) endometrium. PNX-14 was mostly detected in the cytoplasm of stromal cells. GPR173 was noted in some eutopic and ectopic stromal cells and eutopic glandular epithelial cells. Interestingly, some stromal cells showed a positive signal of PNX-14 and GPR173, suggesting a possible autocrine effect of PNX-14.

## 4. Discussion

An investigation of PNX in the proper functioning of the HPG axis was initiated by Yosten et al. in 2013 [7]. Researchers hypothesized that PNX might affect the glandular portion of the pituitary gland. Further studies have shown administration of PNX-14 or PNX-20 increases the release of GnRH due to a regulatory effect on the GnRH receptor (GnRH-R). Furthermore, intrathecal administration of phoenixin significantly reduced pain perception in mice injected intraperitoneally with acetic acid. As a corollary, phoenixin may preferentially suppress visceral pain [19].

Reduction of PNX expression in the hypothalamus using small interfering RNA in vivo has been shown to inhibit the onset of estrus in female rats. By reducing the expression of the pituitary GnRH receptor, this suggests a role of PNX in normal ovarian cyclicity [12]. Since endometriosis is an estrogen-dependent disease, GnRH analogs are used in its treatment and work by inhibiting the pituitary-ovarian axis. Moreover, it has been shown that the direct action of gonadotropin-releasing hormone 1 (GnRH I) on endometriotic cells inhibits proliferation and induction of apoptosis [20]. These observations prompted us to investigate the expression of SMIM20, the precursor protein of PNX, and GPR173, the putative PNX receptor, in eutopic and ectopic endometrium. Additionally, we also assessed the level of PNX, FSH, LH and 17β-estradiol in the serum of women diagnosed with ovarian endometriosis and compare it to the level of normal controls.

The receptor for PNX is considered to be GPR173 based on a ligand binding study by Stein et al. 2016 [21]. Interestingly, PNX regulates Oct-1 and C/EBPβ transcription factor activity, essential for regulating GnRH expression. Subsequent research into the role of GPR173 in the hypothalamus has shown that PNX is essential for a healthy estrus cycle. Injection of siRNA targeting GPR173 has been shown to double estrus cycle length in female rats and eliminate the PNX-induced increase in plasma LH concentration [8]. In summary, it is assumed that PNX potentially has two methods of action on the HPG axis. First is a hypothalamic mode of action as PNX may directly trigger the release of GnRH from hypothalamic neurons. Alternatively, the pituitary may directly activate pituitary gonadotrophs via the pituitary portal vessels. In our studies, we found no expression of SMIM20 differences between eutopic and ectopic endometria and a decrease in the expression of *GPR173* in the ectopic endometrium. Surprisingly, the PNX serum level was significantly lower in women with endometriosis compared to non-endometriosis cases. Based on these results, we speculate that reduced serum PNX levels in women with endometriosis may be responsible for the intensified pain perception and hormonal imbalance maintained by the HPG axis. A correlation of phoenixin levels in sera of endometriosis patients with the level of pain would have been performed to confirm this hypothesis. Sadly, this was not possible in the present study since the study was performed on archival material.

Due to the limited numbers of studies concerning the central and peripheral effects of PNX on diminished fertility, these results are difficult to interpret. Due to decreased PNX concentration in the serum, we expect a disturbance of the central control of the sexual cycle. This likely leads to more frequent anovulatory cycles, forming luteinized unruptured follicle functional cysts. This disorder creates improper progesterone levels in phase II of the cycle therefore promoting the development of endometriosis. At the same time, it does not harm the development of endometrial implants residing in the peritoneum or the ovary. In these foci, there is a self-propelling mechanism of estrogen production through the activity of COX-2 in the foci of the ectopic endometrium. The negative influence on the cycle diminishes the protective effect of progesterone, and at the same time, the ectopic focus of the endometrium retains its autonomy. Furthermore, it is likely that in women with stage III or IV endometriosis, the control axis of GnRH release under PNX is simply disabled. This led us to perform an evaluation of the association of the hormonal profile of FSH, LH and 17β-estradiol with PNX in the blood serum of the studied patients. The analysis revealed an increased LH to FSH ratio and 17β-estradiol levels in the serum of women with endometriosis. Discriminant analysis showed that the LH/FSH ratio and the level of PNX can be used as an algorithm for the non-invasive procedure for detection of ectopic endometrial foci. As mentioned previously, PNX is necessary to maintain cyclicity of both ovaries and thus endometrial changes. The absence of PNX effects results in the impaired release of GnRH. Presumably, in the absence of GnRH in cells forming the ectopic focus, a mechanism initiating intracellular signaling events, such as modulation of transcription factors regulating SMIM20 and GPR173 gene expression, is triggered. Another hypothesis that may explain the decreased PNX expression in serum of women with endometriosis is that GPR173 is not the unique receptor for PNX binding. PNX may be a ligand for an unidentified membrane receptor, not excluding GnRH-R itself. This assumption is the most appropriate and consistent with the studies performed in rats. The authors explain the down-regulation of GPR173 and the increased level of SMIM20 by the existence of molecular interactions between GnRH receptors and PNX signaling in the HPG axis of female rats during the reproductive cycle [12].

Based on immunohistochemical studies, no difference in staining intensity was found between the eutopic and ectopic endometrium. Positive staining for GPR173 was found in eutopic endometrial glands and several stromal cells. In ectopic endometrium, the localization of the examined receptor was restricted to only some of the stromal cells. Remarkably, some fibroblasts in the studied endometria showed a positive signal not only from GPR173 but also from PNX, suggesting the possibility of an autocrine mechanism of PNX action. On the other hand, in the case of SMIM20, expression was mainly confined to stromal cells. This is the first publication presenting data on tissue-specific localization and expression of SMIM20, the precursor protein of PNX-14, and its receptor, GPR173, in the eutopic and ectopic endometrium. The specificity of transcript localization requires further research**.** Moreover, decreased serum PNX-14 concentration in patients with endometriosis suggests the role of PNX-14 in disease pathogenesis as well as in enhancing pelvic pain associated with cyclic changes within the ectopic endometrium.

These new insights may provide not only a better understanding of endometriosis pathophysiology but also lay the potential groundwork for the diagnosis and treatment of endometriosis by targeting SMIM20/PNX. It is worth mentioning that GnRH analogs are often used in the treatment of endometriosis, inducing a menopause-like condition, thus creating side effects that discourage patients from therapy continuation. Perhaps agonists and/or antagonists of PNX or the GPR173 receptor could be used in the future to alter GnRH neuronal function to completely cure endometriosis.

## Figures and Tables

**Table 1:** Primers sequences.

Gene Symbol	NCBI Accession N°	Orientation	Primer Sequence 5′ →3′
*SMIM20*	NM_001145432.2	F	CGGCTTCATCTCCCTGATCG
		R	ACAGCCCTCTCATTTCCTGC
*GPR173*	NM_018969.6	F	CCCGGGCTGTGATTTACCTG
		R	TCCTGCTACATTGCACCTTGG
*B2M*	NM_004048.4	F	GATGAGTATGCCTGCCGTGT
		R	CTGCTTACATGTCTCGATCCCA
*GAPDH*	NM_002046.7	F	CGCTCTCTGCTCCTCCTGTT
		R	CCATGGTGTCTGAGCGATGT
*HPRT1*	NM_000194.3	F	TGACCTTGATTTATTTTGCATACC
		R	CGAGCAAGACGTTCAGTCCT

Legend: F—forward primer; R—Reverse primer.

**Table 2:** The Mann–Whitney U test results for serum PNX, FSH, LH and 17β-estradiol levels.

	Rank Sum (Controls)	Rank Sum (Cases)	Controls (N)	Cases (N)	*p*-Value
*PNX [pg/mL]*	942	598	23	32	**<0.001**
*FSH [mIU/mL]*	400	590	19	25	>0.05
*FSH [mIU/mL]*	381	609	19	25	>0.05
*17β-estradiol [pg/mL]*	304	686	19	25	**<0.01**
*LH/FSH*	294	696	19	25	**<0.01**

Legend: N—number of patients; statistically significant values are in bold.

**Table 3:** Predictors differentiating sera of women with endometriosis from healthy patients.

	Wilks `Lambda	F	*p*-Value
*PNX14 [pg/mL]*	0.57	30.2	<0.001
*LH/FSH*	0.51	19.4	<0.001

Legend: F—value for Fisher’s linear discriminant analysis.

**Table 4:** The Mann–Whitney U test results for normalized Cr data of analyzed genes.

	Rank Sum (Controls)	Rank Sum (Cases)	Controls (N)	Cases (N)	*p*-Value
*GPR173*	2713.5	2746.5	45	59	**<0.05**
*SMIM20*	2459.5	3426.5	46	62	>0.05

Legend: N—number of patients; statistically significant values are in bold.

## Data Availability

The datasets used and analyzed during the current study are available from the corresponding author on reasonable request.
